# miR-300/FA2H affects gastric cancer cell proliferation and apoptosis

**DOI:** 10.1515/med-2020-0188

**Published:** 2020-09-14

**Authors:** Bo Hong, Jie Li, Chunxiao Huang, Tao Huang, Mengpei Zhang, Lijiang Huang

**Affiliations:** Department of Gastroenterology, Xiangshan Hospital Affiliated to Wenzhou Medical University, 291 Donggu Road, Dandong Street, Xiangshan County, Ningbo, 315700, People’s Republic of China

**Keywords:** microRNA-300, fatty acid 2-hydroxylase, gastric cancer, cell proliferation, cell apoptosis

## Abstract

MicroRNA (miR/miRNA) expression disorders play a crucial role in the development of gastric cancer (GC). Increasing evidence has indicated that miRNAs participate in the process of numerous cancers. Previous research has demonstrated that miR-300 acts as a cancer-promoting factor or tumor suppressor in a number of tumors. However, to the best of our knowledge, the effects of miR-300 on GC cells remain largely unknown. The present study investigated the effects of miR-300 on GC cells and analyzed its molecular mechanism. First, reverse transcription–quantitative polymerase chain reaction showed that miR-300 expression was increased in GC tissues and cell lines, with the highest expression observed in human gastric cancer cell line AGS. Subsequent results indicated that fatty acid 2-hydroxylase (FA2H) was a target of miR-300. FA2H-plasmid inhibited AGS cell proliferation and induced apoptosis. Finally, miR-300 inhibitor reduced cell proliferation and induced apoptosis, whereby these effects were reversed by FA2H-small interfering RNA. Therefore, the data demonstrated that miR-300/FA2H might be a new potential biomarker and therapeutic target for GC treatment.

## 1 Introduction

Gastric cancer (GC) is a highly common malignant disease. GC currently ranks second among cancer deaths worldwide, and it has been reported to be responsible for 8,00,000 deaths among 1 million new GC cases, but its incidence is decreasing with the progress of society [1]. In addition, the development of GC is affected by human lifestyle behaviors and can be prevented [2]. Studies have shown that primary metastatic tumors cause the majority of cancer patients to die [3]. A large 2018 data set revealed ∼26,240 GCs and 10,800 deaths [4]. Although numerous treatment methods are available that can improve the survival rate of GC, the survival time is still short [5,6,7]. Additionally, effective therapies are limited [8,9]. At present, surgery remains the only curative therapy for GC, while perioperative and adjuvant chemotherapy, as well as chemoradiation, can improve the outcome of resectable GC with extended lymph node dissection [10,11]. However, more than half of the radically resected GC patients demonstrated relapse locally or with distant metastases [11]. Therefore, it is necessary to find a new therapeutic target and better understand the molecular mechanisms of GC development.

MicroRNAs (miRs/miRNAs) are a group endogenous, noncoding, and single-stranded small molecule RNAs. The miRNAs contain ∼18–24 nucleotides that mediate downstream gene expression at the posttranscriptional level [12,13,14]. The miRNAs participate in a number of biological processes, and the miRNA expression and functional changes are associated with numerous diseases [15]. More studies suggested that miRNAs were abnormally expressed in multiple developmental processes of GC [16,17]. Li et al. showed that miR-300 regulates inflammatory responses through the activation of AMPK/mTOR signaling pathway in neonatal sepsis [18]. Zhou et al. demonstrated that miR-300 serves as a potential biomarker for osteoarthritis patients [19]. Liu et al. indicated that serum miR-300 might act as a diagnostic and prognostic biomarker in osteosarcoma [20]. He et al. showed that miR-300 regulates cellular radiosensitivity in human lung cancer cells [21]. Interestingly enough, a very recent publication reported the upregulation of miR-300 in GC, providing potential therapeutic targets for clinical data [22]. Recent studies showed that miR-300 has a cancer-promoting effect on numerous tumors, but its effect on GC cells has not been fully elucidated. The present study focused on investigating the effects of miR-300 on the proliferation and apoptosis of GC cells and analyzing its molecular mechanism.

As a hydroxy fatty acid enzyme, fatty acid 2-hydroxylase (FA2H) can promote 2-hydroxylation of fatty acid *N*-acyl chains [23]. It was reported that 2-hydroxyceramide and FA2H, which are present in various organs [24,25], participate in cell signaling pathways. In addition, FA2H participates in tumor progression [26]. Previous research indicated that FA2H can affect the cell cycle and cell migration and promote the sensitivity of tumor cells to drugs and regulate the drug resistance of tumor cells. Yao et al. demonstrated that FA2H can inhibit the growth of GC cells and increase their sensitivity to drugs [26]. FA2H catalyzes the introduction of a chiral (R)-hydroxy group at the second carbon atom of long-chain fatty acids, resulting in the formation of (R)-2-OHFAs [27]. The effects of (R)-2-OHPA treatment alone in tumor suppression *in vivo* were minimal, while FA2H knockdown significantly enhanced tumor growth [27]. These results raise the issue of possible involvement of other (R)-2-OHFAs produced by FA2H. However, to the best of our knowledge, the specific roles of FA2H and 2-hydroxy fatty acids as participants in nutritional metabolism in regulating tumors and their mechanisms have not been fully elucidated.

Using bioinformatics software analysis, it was found that miR-300 and FA2H have direct binding sites. Therefore, it was hypothesized that miR-300 may regulate GC cell function by regulating FA2H expression.

## 2 Materials and methods

### 2.1 Tissue samples and cell culture

A total of 38 samples of GC tissues and adjacent tissues were collected to perform the following experiments. GC tissue samples were snap frozen in liquid nitrogen and stored at −80°C for reverse transcription–quantitative polymerase chain reaction (RT-qPCR) analysis. The present study protocol was approved by the Ethical Review Committee of Xiangshan Hospital Affiliated to Wenzhou Medical University. Each patient provided written informed consent.

GC cell lines (AGS, SNU-1, SNU-5, and NCIN87) and the normal gastric mucosal epithelial cell line GES-1 were acquired from the Chinese Academy of Sciences. All cell lines were cultured in RPMI-1640 medium (Gibco; Thermo Fisher Scientific, Inc.) supplemented with 10% fetal bovine serum (Gibco; Thermo Fisher Scientific, Inc.) and incubated at 37°C in a 5% CO_2_ incubator.

### 2.2 Cell transfection

AGS cells were transfected with 1 µg control plasmid (Cat no. sc-437275; Santa Cruz Biotechnology), 1 µg FA2H lasmid (Cat no. sc-413143-ACT; Santa Cruz Biotechnology), 0.2 µM control small interfering RNA (siRNA; Cat no. sc-36869; Santa Cruz Biotechnology), 0.2 µM FA2H siRNA (Cat no. sc-93418; Santa Cruz Biotechnology), 100 nM inhibitor control (5′-CAGUACUUUUGUGUAGUACAA-3′; GenePharma, Shanghai, China), 100 nM miR-300 inhibitor (5′-AGAGAGAGUCUGCCUUGUAUA-3′; GenePharma), 100 nM miR-300 inhibitor + 0.2 µM control siRNA or 100 nM miR-300 inhibitor + 0.2 µM FA2H siRNA for 48 h with Polyplus transfection reagent (Invitrogen; Thermo Fisher Scientific, Inc.), according to the manufacturers’ instructions before subsequent experimentation.

### 2.3 RT-qPCR

Total RNA was acquired using TRIzol (Takara Bio, Inc.), according to the manufacturer’s instructions. Successful RNA extraction was determined by the presence of three bands on the nucleic acid gel. Once the RNA extraction was successfully performed.

The RNA was reverse transcribed into cDNA using a reverse transcription kit (Vazyme Biotech Co., Ltd). Subsequently, the cDNA was used for amplification. qPCR was performed using a SYBRGreen PCR kit (Vazyme Biotech Co., Ltd), according to the manufacturer’s instructions. GAPDH (for mRNA) or U6 (for miRNA) was used as endogenous controls. Primer sequences were listed as following:

miR-300 forward, 5′-TATACAAGGGCAGACTCTCTCT-3′;

reverse, 5′-CGCAAGGATGACACGCAAATTCGT-3′;

GAPDH forward, 5′-CTTTGGTATCGTGGAAGGACTC-3′;

reverse, 5′-GTAGAGGCAGGGATGATGTTCT-3′;

U6 forward, 5′-GCTTCGGCAGCACATATACTAAAAT-3′；and

reverse, 5′-CGCTTCACGAATTTGCGTGTCAT-3′.

The 2^−ΔΔCq^ method was used to quantify relative gene expression. All samples were performed in triplicate, and all experiments were repeated three times.

### 2.4 Western blot assay

Cells were lysed, and the total protein was obtained using RIPA buffer (Beyotime Institute of Biotechnology). A bicinchoninic acid assay kit (Pierce; Thermo Fisher Scientific, Inc.) was used to quantify the total protein. Equal amounts of protein were separated by 12% SDS-PAGE for 40 min and then transferred to PVDF membranes (EMD Millipore). The membranes were blocked for 1.5 h at room temperature with 5% nonfat milk and incubated with primary antibodies including anti-FA2H (Cat no. ab128917; 1:1,000; Abcam), anticleaved caspase-3 (Cat no. ab32042; 1:1,000; Abcam), and anti-pro-caspase-3 (Cat no. ab32499; 1:1,000; Abcam) overnight at 4°C. The next day, the membranes were incubated with horseradish peroxidase-conjugated antirabbit secondary antibody (Cat. no. 7074; 1:2,000; Cell Signaling Technology, Inc.) for 2 h. Protein bands were visualized by enhanced chemiluminescence (GE Healthcare Life Sciences). β-Actin (1:1,000; Abcam) served as the loading control for normalization.

### 2.5 Flow cytometry assay

Cell apoptosis was assessed using the Annexin-V/propidium iodide (PI) apoptosis detection kit. Briefly, the cells were plated in six-well plates at a density of 2–3 × 10^5^ cells/well overnight. The next day, specific inhibitor, plasmid, or siRNA was transfected into the AGS cells. The cells were then directly collected, centrifuged in low temperature at high speed and resuspended in 100 µL FITC-binding buffer. Subsequently, ∼5 µL ready-to-use Annexin V-FITC (BD Biosciences) and 5 µL PI were added into the buffer. The cells protected from light were incubated for 30 min at room temperature. Annexin V-FITC and PI fluorescence were assessed using a BD FACSCalibur flow cytometer (BD Biosciences).

### 2.6 Dual-luciferase reporter assay

The wild-type (WT) or mutant (MUT) 3′-untranslated region (3′-UTR) of FA2H was cloned into a pmiRGLO vector (Promega Corporation). Recombinant plasmids were acquired using an EndoFree Plasmid Maxi kit (Vazyme Biotech Co., Ltd). A total of 293 T cells seeded in 24-well plates were cotransfected with miR-300 mimics (sense: 5′-UAUACAAGGGCAGACUCUCUCU-3′, antisense: 5′-AGAGAGUCUGC CCUUGUAUAUU-3′; GenePharma) or negative control (sense: 5′-UUCUCCGAACGUGUCACGUTT-3′, antisense: 5′-ACGUGACACGUUCGG AGAATT-3′; GenePharma) and the MUT or WT 3′-UTR of FA2H for 48 h together with renilla luciferase pRL-TK vector as a control. Following transfection for 48 h, firefly and renilla luciferase activities were tested using a dual-luciferase reporter assay (Promega Corporation). Firefly luciferase activity was normalized to renilla luciferase activity.

### 2.7 MTT assay

Cell viability was measured using an MTT assay. AGS cells were plated into a 96-well plate and then incubated for 24, 48, or 72 h. Subsequently, 20 µL MTT solution (5 mg/mL; Sigma-Aldrich; Merck KGaA) were added into each well and further cultured for another 4 h. The absorbance was measured at a wavelength of 570 nm. Data were analyzed as the mean ± standard deviation (SD) of three separate experiments.

### 2.8 Statistical analysis

Statistical analyses were performed using GraphPad Prism 6 software (GraphPad Software, Inc.). Statistical significance differences between groups were determined by Student’s *t* test or analysis of variance with Tukey’s *post hoc* tests. Data were expressed as the mean ± SD from at least three independent experiments. *P* < 0.05 was considered to indicate a statistically significant difference.

## 3 Results

### 3.1 Expression levels of miR-300 in GC tissues and cells

**Figure 1:**
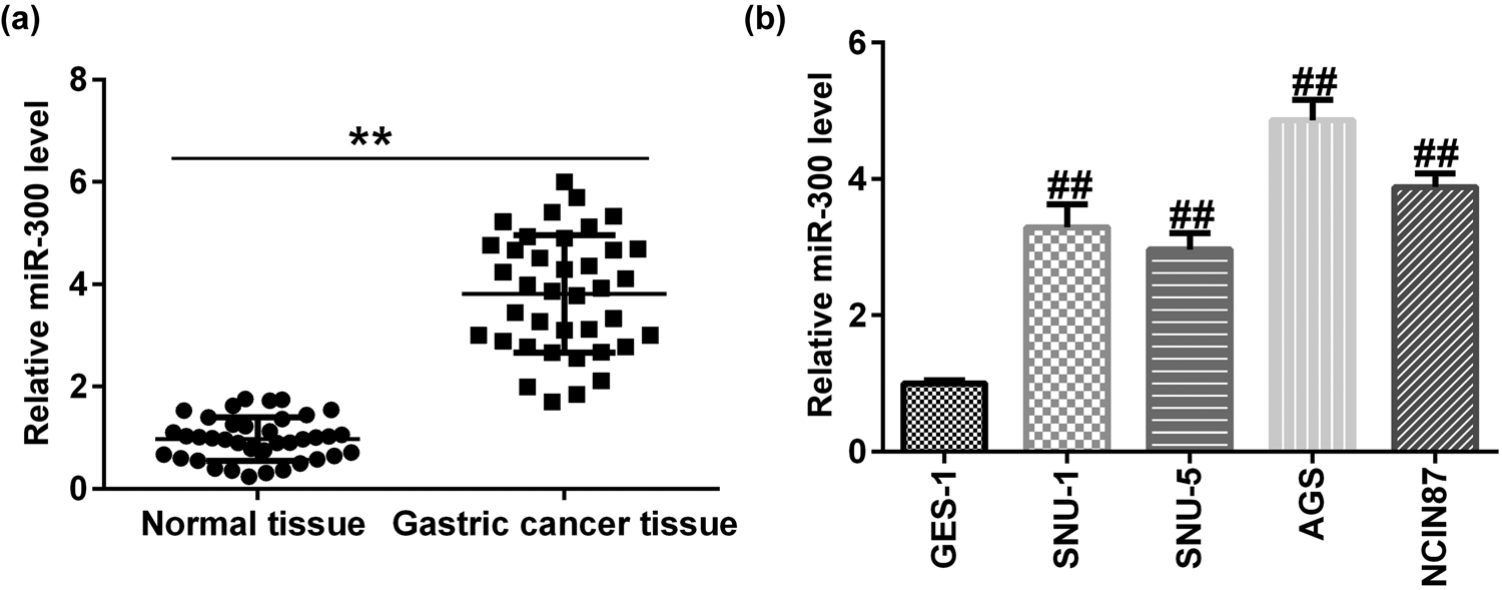
miR-300 is upregulated in GC. Reverse transcription–quantitative PCR assay detected the relative expression of miR-300 in (a) GC and adjacent tissues and (b) GC cell lines. miR, microRNA; GC, gastric cancer.

To investigate the role of miR-300 in GC, the GC and adjacent tissues in 38 patients were examined using RT-qPCR analysis, which indicated that miR-300 was significantly upregulated in GC tissues compared with the matched adjacent normal tissues (Figure 1a). Furthermore, miR-300 expression levels were measured in different GC cell lines. miR-300 was positively expressed in AGS, SNU-1, SNU-5, and NCIN87 cells compared with nonmalignant GC cell line GES-1 (Figure 1b). The highest expression was found in AGS cells.

### 3.2 FA2H is a target gene of miR-300

**Figure 2:**
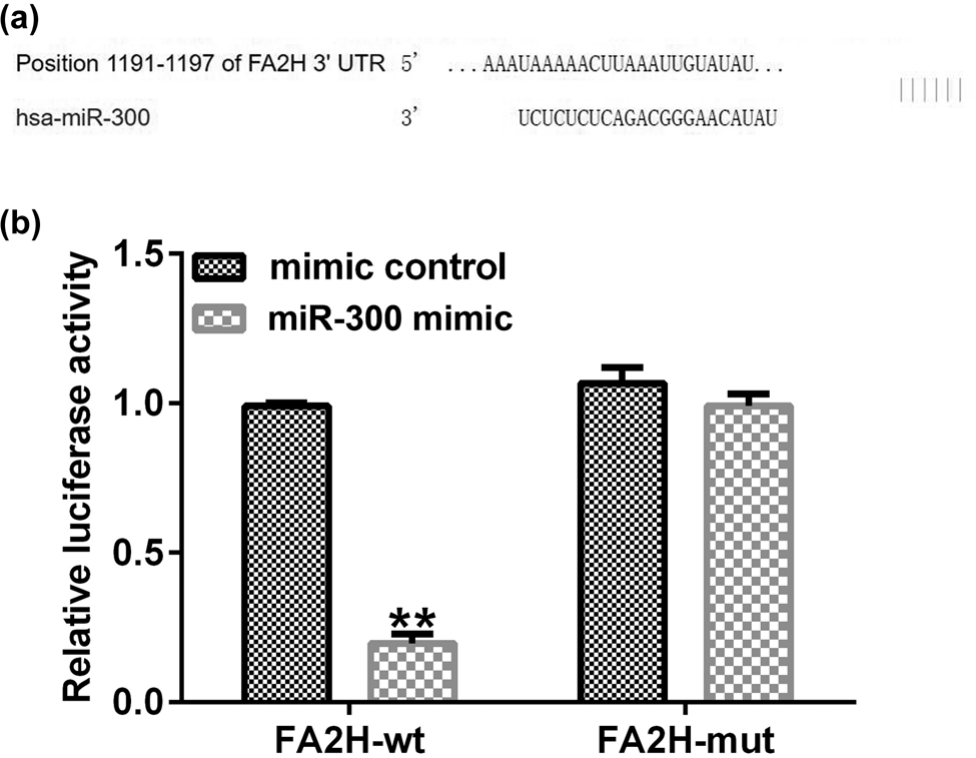
FA2H is a target gene of miR-300. (a) Prediction of miR-300 targeting FA2H using the miR target gene database TargetScan. (b) Dual luciferase reporter gene assay verifying the relationship between miR-300 and FA2H in 293T cells transfected with miR-300 mimic and WT or mutant FA2H 3′-UTR reporter. FA2H, fatty acid 2-hydroxylase; miR < microRNA; 3′-UTR, 3′-untranslated region.

To study the underlying mechanism, the bioinformatics prediction algorithm TargetScan was used. The results showed that FA2H may be a target gene downstream of miR-300 (Figure 2a). Subsequently, a dual-luciferase assay was performed to confirm their relationship. The 3′-UTR (either WT or MUT) of FA2H was inserted into a pmiR luciferase reporter, and 293T cells were cotransfected with miR-300 mimic or mimic control and FA2H-WT or FA2H-MUT. In addition, the results indicated that miR-300 mimic cotransfection with WT FA2H 3′-UTR reporter inhibited luciferase activity, but miR-300 mimic did not exert effects on the MUT-containing reporter (Figure 2b).

### 3.3 Effects of FA2H on the proliferation and apoptosis of AGS cells

**Figure 3:**
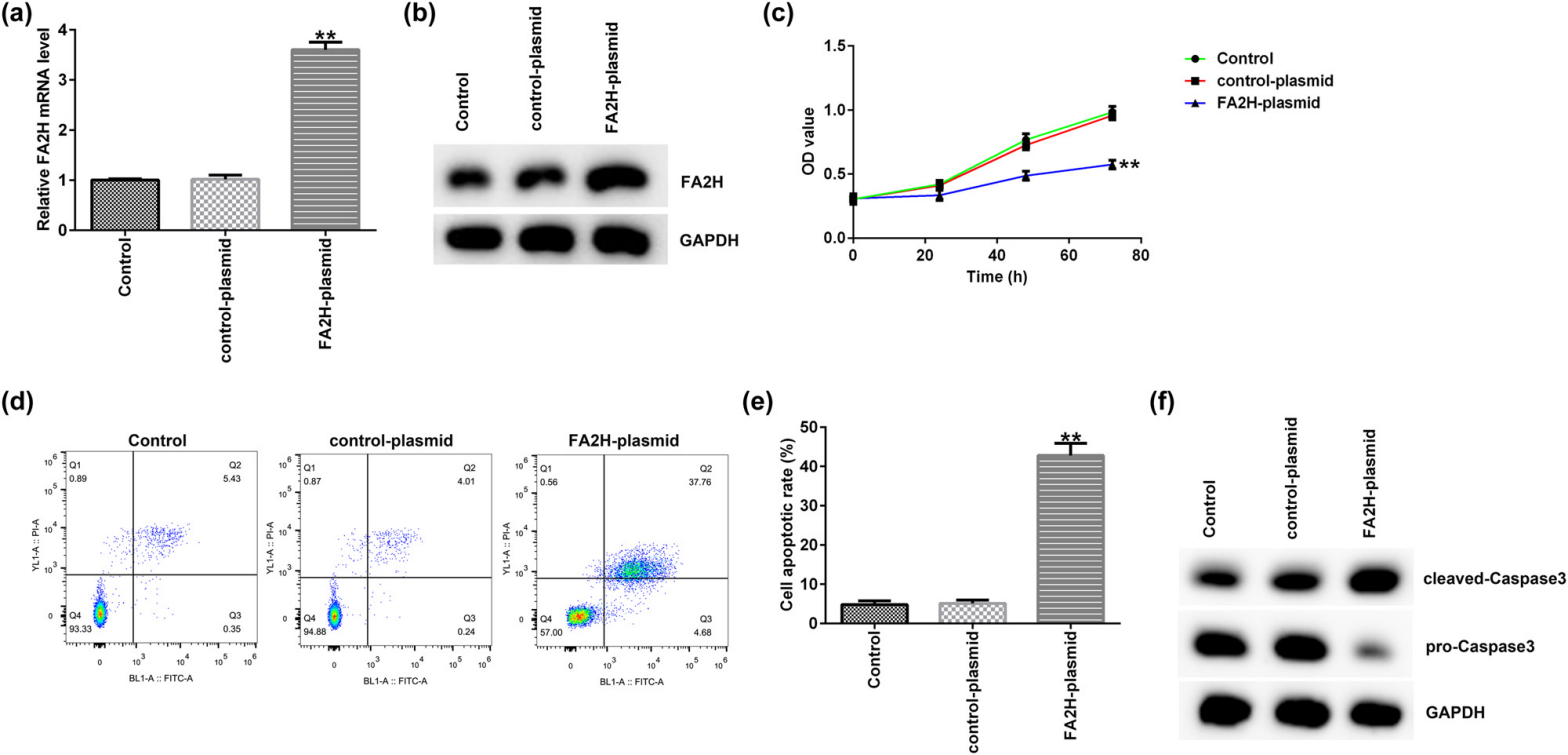
FA2H plasmid decreases AGS cell proliferation and promotes apoptosis. (a) Reverse transcription–quantitative PCR assay detected relative FA2H expression in AGS cells transfected with FA2H plasmid. (b) Western blot assay detected FA2H expression at the protein level. (c) MTT assay detected cell proliferation in AGS cells transfected with FA2H plasmid for 48 h. (d) Flow cytometry assay analyzed cell apoptosis. (e) Cell apoptosis rates. (f) Western blot assay detected cleaved caspase-3 and pro-caspase-3 expressions in AGS cells. FA2H, fatty acid 2-hydroxylase.

Subsequently, the effects of FA2H on the proliferation and apoptosis of AGS cells were assessed. Control plasmid or FA2H plasmid was transfected into AGS cells for 48 h. Both RT-qPCR and Western blot assay showed that compared with the control plasmid group, FA2H plasmid significantly increased FA2H expression in the AGS cells (Figure 3a and b). The MTT assay analysis indicated that FA2H plasmid reduced the cell proliferation of GC cell AGS (Figure 3c). Flow cytometry assay demonstrated that FA2H plasmid induced cell apoptosis (Figure 3d and e). Furthermore, FA2H plasmid significantly increased the cleaved caspase-3 protein and decreased the pro-caspase-3 protein expression (Figure 3f).

### 3.4 Effects of low miR-300 expression on the proliferation and apoptosis of AGS cell by increasing FA2H expression

**Figure 4:**
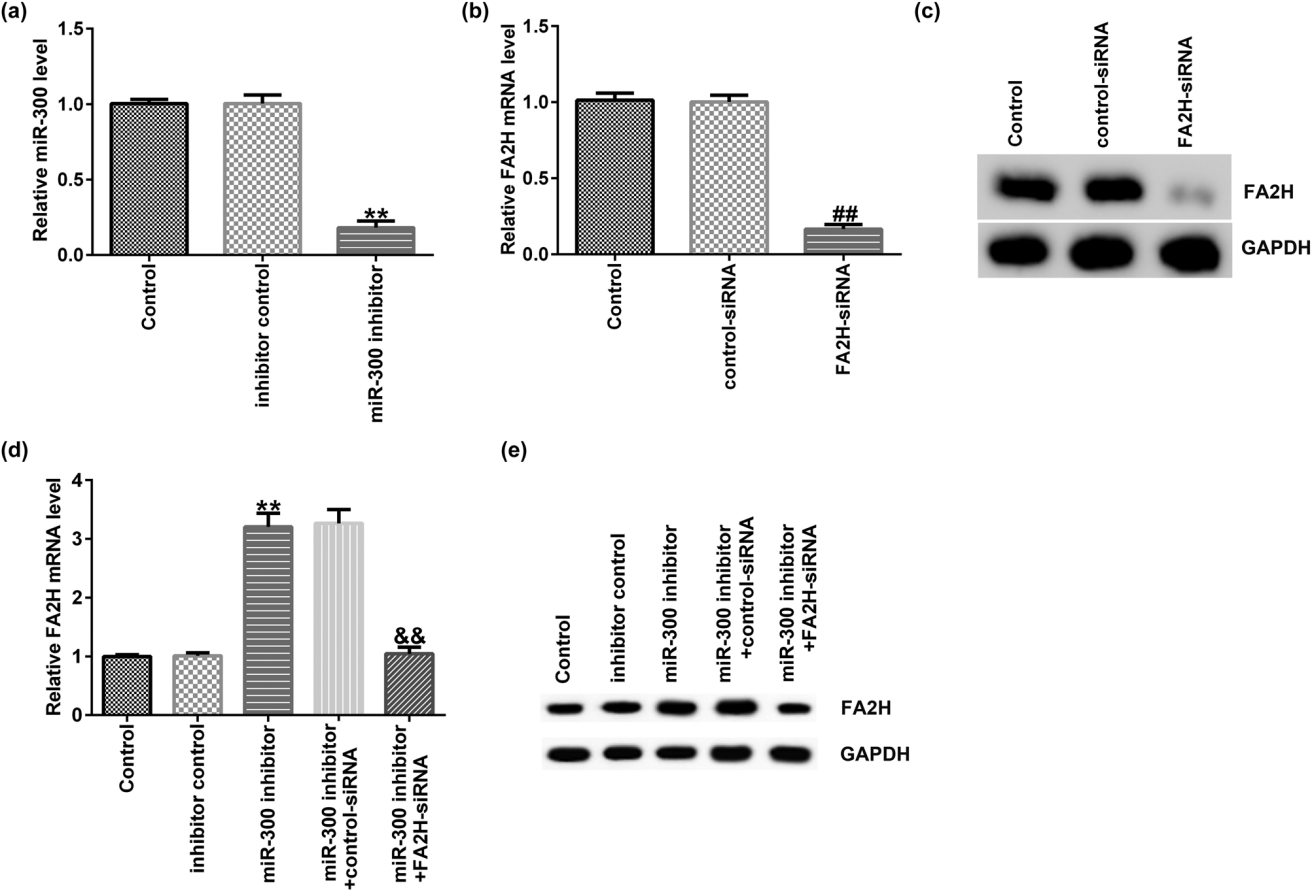
miR-300 negatively regulates FA2H expression in AGS cells. (a) RT-qPCR assay detected miR-300 expression in AGS cells transfected with inhibitor control or miR-300 inhibitor. (b and c) RT-qPCR assay and Western blot assay detected FA2H mRNA expression in AGS cells transfected with control siRNA or FA2H siRNA. (d and e) RT-qPCR assay and Western blotting detected FA2H mRNA and protein expression in AGS cells transfected with inhibitor control, miR-300 inhibitor, miR-300 inhibitor + control siRNA, or miR-300 inhibitor + FA2H siRNA. miR, microRNA; RT-qPCR, reverse transcription-quantitative PCR; FA2H, fatty acid 2-hydroxylase; siRNA, small interfering RNA.

First, AGS cells were transfected with control siRNA, FA2H siRNA, inhibitor control, miR-300 inhibitor, miR-300 inhibitor + control siRNA, or miR-300 inhibitor + FA2H siRNA for 48 h. The following experiments were then performed. Compared with the inhibitor control group, miR-300 inhibitor significantly reduced miR-300 expression in AGS cells (Figure 4a). RT-qPCR demonstrated that in comparison with the control siRNA group, FA2H siRNA significantly reduced FA2H expression in AGS cells (Figure 4b and c). Compared with the inhibitor control group, miR-300 inhibitor significantly increased FA2H expression in AGS cells, and this increase was reduced by FA2H siRNA (Figure 4d and e).

**Figure 5:**
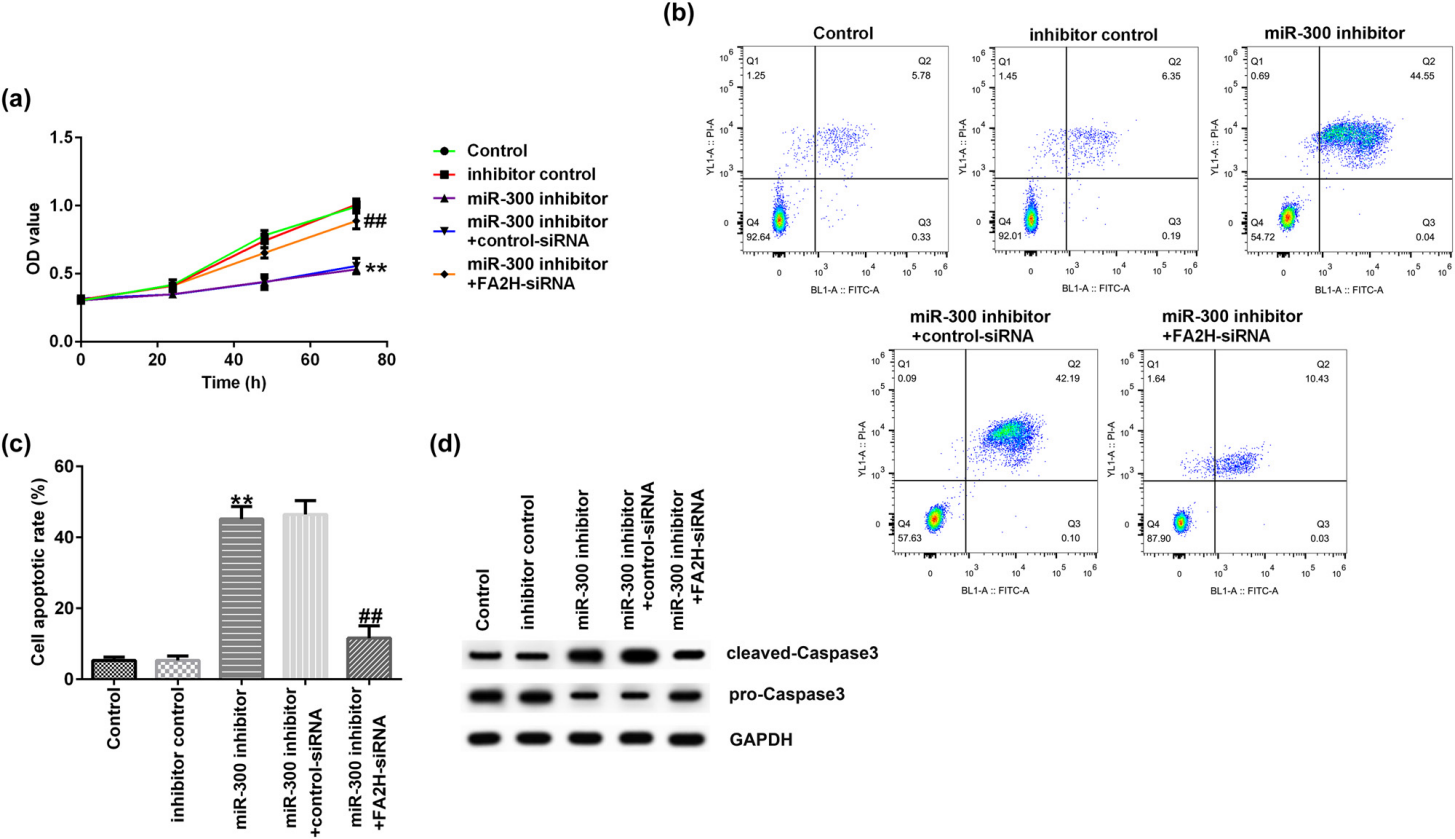
miR-300 inhibitor reduces AGS cell proliferation, the effects of which are reversed by FA2H siRNA. AGS cells were transfected with inhibitor control, miR-300 inhibitor, miR-300 inhibitor + control siRNA, or miR-300 inhibitor + FA2H siRNA for 48 h. (a) MTT assay showing cell proliferation. (b) Flow cytometry assay showing cell apoptosis. (c) Cell apoptosis rates. (d) Western blot assay showing cleaved caspase-3 and pro-caspase-3 expressions in AGS cells. miR, microRNA; FA2H, fatty acid 2-hydroxylase; siRNA, small interfering RNA.

Finally, MTT and flow cytometry assays showed that compared with the inhibitor control group, miR-300 decreased cell proliferation (Figure 5a) and induced cell apoptosis (Figure 5b and c). These changes were all reversed by FA2H siRNA. Western blot assay showed that miR-300 increased cleaved caspase-3 protein and reduced pro-caspase-3 protein expressions (Figure 5d), and these changes were reversed by FA2H siRNA transfection.

## 4 Discussion

With high mortality rates reported each year, GC has posed a burden on global public health. Since the early stage patients with GC typically show no symptoms, numerous patients are diagnosed with advanced GC upon going to hospital to seek help for stomach problems. Late stage patients with GC have low survival rates due to tumor metastasis. Therefore, postoperative care, nutrition diet, and relaxation should be improved.

There is an urgent need to discover new miRNAs, which will explore therapeutic targets to overcome drug resistance [28]. miRNAs could regulate different biological processes of tumorigenesis, including the conversion of primary cells, tumor cell proliferation, invasion, metastasis, and the occurrence of epithelial–mesenchymal transition [29]. For example, miR-215 promoted cell migration and invasion of GC by targeting retinoblastoma tumor suppressor gene 1 [30]. Zhang et al. showed that miRNA-574-5p promoted angiogenesis via tyrosine-protein phosphatase nonreceptor type 3 in GC [31]. Wei et al. demonstrated that miR-638 regulated cell proliferation via targeting metastasis-associated colon cancer protein 1 [32]. Furthermore, miR-300 is associated with multiple tumors such as bladder cancer [33], colorectal carcinoma [34], breast cancer [35], lung cancer [36], and hepatocellular carcinoma [37]. However, to the best of our knowledge, few studies have reported the effects of miR-300 on GC cells. The present study aimed to explore the effects of miR-300 on the proliferation and apoptosis of GC cells.

The present study found that miR-300 was upregulated in tissues and cell lines in GC, and the miR-300 downregulation inhibited cell proliferation and induced apoptosis in AGS cells. Subsequently, TargetScan software was used and experiments were performed to confirm whether FA2H was a target of miR-300. FA2H is a chiral (R)-hydroxyl group inserted at the second carbon of long-chain FA. It was reported that FA2H was overexpressed in a number of organs. In addition, FA2H also affects cell differentiation and regulates the membrane transport capacity of nutrient transporters [38,39]. Alderson et al. demonstrated that FA2H silencing promoted D6P2T nerve sheath cell proliferation and suppressed cAMP-induced cell cycle arrest [40], suggesting that FA2H has multiple functions in regulating signaling pathways associated with cell proliferation. The present results showed that low expression of miR-300 affected the proliferation and apoptosis of AGS cells by increasing the expression of FA2H. However, in this study, only one type of siRNA of FA2H was used to knockdown FA2H expression in AGS cells; and to make the results more convincing, multiple siRNA of FA2H or different approach like CRISPR knockout need to be provided. In addition, we tested the transfection efficiency of FA2H siRNA only 48 h after cell transfection. These might be the limitations of this study.

In conclusion, miR-300 downregulation inhibited GC cell proliferation and induced apoptosis in an FA2H-dependent manner. Therefore, miR-300/FA2H might be a new potential biomarker and therapeutic target for GC treatment. However, this study was only a preliminary *in vitro* study of the role of miR-300 in GC. To make the role of miR-300 in GC more clear and credible, a lot of in-depth research is needed. For example, the role of miR-300/FA2H in other GC cell lines needs to be elucidated. The effect of miR-300/FA2H on GC should be investigated *in vivo*. Moreover, whether there is any correlation of the level of expression of miR-300/FA2H with the patients’ pathological or even demographic data should be explored. We will perform these issues in the future.
